# Radiologic predictors for failure of non-operative management of complicated diverticulitis: a single-centre cohort study

**DOI:** 10.1007/s00423-021-02244-3

**Published:** 2021-06-29

**Authors:** Stefan Reischl, Kai Dominik Roehl, Sebastian Ziegelmayer, Helmut Friess, Marcus Richard Makowski, Dirk Wilhelm, Alexander Rudolf Novotny, Jochen Gaa, Philipp-Alexander Neumann

**Affiliations:** 1 School of Medicine, Department of Surgery, Technical University of Munich, Munich, Germany; 2 School of Medicine, Department of Diagnostic and Interventional Radiology, Technical University of Munich, Munich, Germany

**Keywords:** Acute diverticulitis, Contained perforation, Resection, Conservative treatment, Prediction

## Abstract

**Purpose:**

Modern non-operative management of diverticulitis consists of a complex therapeutic regimen and is successful in most cases even of complicated diverticulitis. Still, a certain proportion of patients requires urgent surgery due to failure of the conservative approach. This study aims to identify predictors for failure of conservative treatment of complicated diverticulitis with the need for subsequent urgent resection during the acute episode.

**Methods:**

A single-centre retrospective cohort study was performed at our tertiary centre including cases of acute complicated diverticulitis (characterized by localized abscess formation and/or pericolic air) between 2007 and 2019 that were treated guideline-conform by multimodal conservative treatment. Radiologic characteristics of disease in CT scans upon admission were analysed by uni- and multivariable logistic regression to determine predictors for resection within 30 days after onset of the conservative therapy approach.

**Results:**

A total of 669 cases of acute diverticulitis were identified, of which 141 patients met the inclusion criteria. Overall, 13% (*n* = 19) of patients were operated within 30 days despite initial conservative management. Multivariable logistic regression identified length of inflamed bowel greater than 7 cm (*p* < 0.011) and abscess formations >1 cm (*p* < 0.001) as significant risk factors for failure of conservative treatment.

**Conclusion:**

Patients with length of inflamed bowel >7 cm or abscess formation >1 cm have increased risk for failure of conservative treatment of acute episodes of diverticulitis with contained perforations with subsequent need for urgent surgery. Therefore, conservative treatment of those patients should be monitored with special caution.

## Introduction

Acute diverticulitis is a common and but potentially life-threatening inflammatory disease mostly of the sigmoid or descending colon of the older population. Clinical practice guidelines have gone through multiple changes during recent years [1, 2]. While cases with free perforations and generalized peritonitis require emergency surgery [3], in cases with contained perforation, represented by localized intraabdominal air or abscess formation, the approach is primarily conservative. Modern conservative management is a complex approach basing on the three main pillars ‘dietary restrictions’, ‘antibiotic treatment’ and ‘interventional drainage’. Although about 80% of conservative treatment approaches for acute presentations of diverticulitis are successful, patients with contained perforations are a subpopulation with a high risk of failure of the non-operative approach [4]. In case of failure, urgent resection of the affected bowel segment can become necessary to avoid severe septic courses. In those cases, surgical management might be even more challenging than immediately at admission due to potential septic progression, and bearing a higher risk of postoperative complications. Although there are known risk factors for successful conservative management of patients with acute complicated diverticulitis, further early and especially individual determinants for failure of conservative treatment are still urgently needed to optimize medical management of complicated diverticulitis [5].

Studies have shown that findings of paracolic fluid collection, colonic fistula formation, extraluminal air and bowel obstruction in CT scans were able to predict the need for surgical management at any time point [6, 7]. However, data on additional quantitative interpretation of CT findings to predict disease outcomes and particularly the need for early surgery despite an initial conservative approach is scarce. To facilitate the decision for early surgical resection, quantification of additional markers for disease severity in CT could have a high prognostic value. Still, to the best of our knowledge, no study performed a systematic approach to address this question.

The aim of this study is to find quantitative predictors for resection within 30 days in patients suffering from acute diverticulitis with contained perforations that were initially selected for a conservative approach and in which the aim to reach the inflammation free interval for elective surgery was missed. We hypothesize that in addition to established radiologic and clinical determinants, further quantitative radiologic markers might have a high predictive value on the need for surgical resection.

## Materials and methods

The study was designed as a single-centre retrospective cohort study at our tertiary institution. It was approved by the institutional ethical board of the Technical University of Munich (No. 10/19s). Informed consent was waived according to the regulations of our university for retrospective analyses.

### Patient cohort and conservative treatment regimen

The patient cohort consisted of patients with diagnosis of acute diverticulitis complicated by localized intraabdominal abscess formation with pericolic or pelvic localization (not generalized peritonitis) and/or localized pericolic extraluminal air (not distant intraabdominal air), referred as ‘contained perforations’ in the following. There are multiple inhomogeneous and nation-dependent classification systems for acute diverticulitis. Our cohort consisted of patients with signs of (contained) perforation and therefore roughly corresponds to modified Hinchey classification Ib and II [8]. These patients should be treated conservatively according to current guidelines but still have a high probability for failure of conservative management. Treatment of the included patients at our centre adheres to current guideline recommendations [9]. Modern conservative management of those patients is a complex regimen consisting of several main pillars: empiric intravenous broad-spectrum antibiotic treatment, interventional drainage of abscesses >4 cm and dietary restrictions during the acute episode potentially complemented by parenteral nutrition. The decision for necessity of operative treatment despite initial conservative approach was made by the consultant surgeon in charge at the ward at our hospital. The decision for urgent surgery for failure of non-operative management was made for patients that despite maximal conservative treatment did not clinically improve or even deteriorate in condition.

### Inclusion and exclusion criteria

**Fig. 1:**
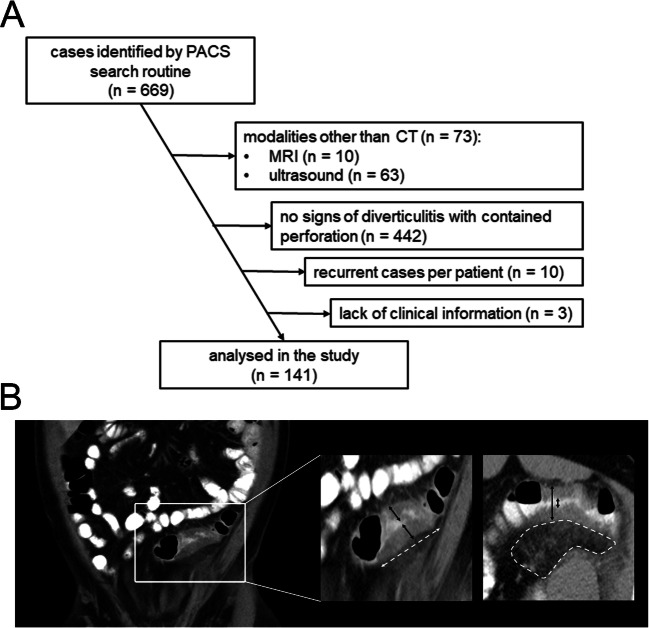
Inclusion flowchart and radiologic measurement methodology. **A** Patient inclusion flowchart. Cases potentially eligible were identified by a PACS search query. Stepwise exclusion was performed to achieve the final cohort analysed in the study. **B** Radiologic measurements are demonstrated in an exemplary CT scan in coronal (left and middle panel) and axial plane (right panel)

The patient cohort was retrieved from the Picture Archiving and Communication System (PACS) of the Department of Radiology at our centre through a search for diverticulitis cases between January 2007 and January 2019. Search terms were ‘diverticulitis’ and ‘perforation’ within the radiologic reports. Patients that were identified by the search routine were then examined for eligibility. To achieve the final cohort, patients were excluded stepwise: First, all cases with modalities other than CT were removed. Next, all cases not being cases of acute diverticulitis with contained perforations (modified Hinchey classification Ib or II) were removed. Third, for multiple recurrent cases of the same patient, only the earliest case treated at our institution was included. Last, cases with significant lack of clinical information were excluded (Fig. 1A).

### Image analysis

CT imaging at the time of initial admission of each included patient at our centre was used for digital image analysis. Image segmentation and quantification routine were designed by a consultant radiologist (20 years of experience) and a consultant surgeon (11 years of experience). Re-evaluation of the CT scans was performed in Sectra IDS7 (Linkoeping, Sweden). Raters were blinded for patient characteristics, initial CT report and patient outcome. Relevant radiologic features to quantify inflammation of the colon and the surrounding mesenteric tissue were examined (Fig. 1B). Measurements were performed in one axial and one coronal section of the CT scan, each showing the highest extent of disease, and the mean value was calculated. The following parameters were measured: length of inflamed bowel segment (in cm), maximum diameter of inflamed bowel segment (in cm), minimal lumen of inflamed bowel segment (in mm), maximal wall thickness of inflamed bowel (in cm), area of mesenteric inflammation (in cm^2^) and size of abscess formation. Two values were indirectly calculated from measurements: Density of mesenteric inflammation (in HU) was calculated by subtracting the density (in HU) of non-inflamed mesenteric tissue from the density of inflamed mesenteric tissue (in HU). Total mesenteric inflammation (in HU * dm^2^) was calculated by multiplication of the values area of mesenteric inflammation and density of mesenteric inflammation. Liquid abscess formations were noted when larger than 1 cm, as below 1 cm a safe radiologic differentiation between liquid abscesses and non-liquefied tissue is not feasible. Additionally, large abscesses >4 cm and the number of interventionally drained abscesses were recorded.

### Clinical data acquisition

The follow-up period started at January 2007 and ended February 2019. For all included patients, relevant clinical data representing outcomes, epidemiological data, risk factors and potential confounders as well as disease-specific data of the included cases were collected (Table 1). Clinical data was extracted from the electronic patient records of the hospital for the initial and follow-up visits in our outpatient clinic. The primary dependent variable was occurrence of surgical resection within 30 days after onset of conservative therapy at admission. This time point was chosen as cut-off, as our institutional policies (which did not change during the inclusion period) recommend the initial conservative management for diverticulitis cases with contained perforation followed by resection no earlier than 6 weeks from the acute episode. Therefore, patients resected within 30 days after initial conservative management represent a failure of the approach for initial conservative management. Clinical variables were sex, age, American Society of Anaesthesiologists physical status classification system (ASA) score, comorbidities, diabetes and medical immunosuppression, leucocyte counts and CRP values at admission, number of episode, type and duration of antibiotic therapy, time between onset of pain and admission and duration of hospital stay.

**Table 1:** Overview of the epidemiologic data, risk factors and disease-specific information of the study population and the subgroups. Data are presented as mean ± standard deviation or patient numbers and percentage of total patients in the cohort or subgroup. *p*-values are indicated behind the groups

	Total	Resection within 30 days	No resection within 30 days	*p*
Total	141 (100%)	19 (13%)	122 (87%)	
Sex * 0.142*				
Male	81 (57.4%)	14 (73.7%)	67 (54.9%)	
Female	60 (42.6%)	5 (26.3%)	55 (45.1%)	
Age (in years)	56.2 14.6	60.7, 12.6	55.5 (14.9)	*0.107*
ASA				***0.001***
I	32 (22.7%)	1 (5.3%)	31 (25.4%)	
II	68 (48.2%)	3 (15.8%)	65 (53.3%)	
III	35 (24.8%)	12 (63.2%)	23 (18.9%)	
IV	6 (4.3%)	3 (15.8%)	3 (2.5%)	
V and VI	0 (0.0%)	0 (0.0%)	0 (0.0%)	
Comorbidities				*0.191*
None	65 (46.1%)	5 (26.3%)	60 (49.2%)	
Multiple	48 (34.0%)	13 (68.4%)	35 (28.7%)	
Cardiovascular	19 (13.5%)	1 (5.3%)	18 (14.8%)	
Others	9 (6.4%)	0 (0.0%)	9 (7.4%)	
Diabetes	5 (3.5%)	0 (0.0%)	5 (4.1%)	*0.614*
Immunosuppression				*0.172*
None	134 (95.0%)	17 (89.5%)	117 (95.9%)	
Biologicals	0 (0.0%)	0 (0.0%)	0 (0.0%)	
Steroids	4 (2.8%)	1 (5.3%)	3 (2.5%)	
Immunomodulators	1 (0.7%)	1 (5.3%)	0 (0.0%)	
Cytostatic drugs	2 (1.4%)	0 (0.0%)	2 (1.6%)	
Time between pain onset and admission				***0.007***
< 24 h	45 (37.2%)	4 (33.3%)	41 (37.6%)	
24–72 h	30 (24.8%)	2 (16.7%)	28 (25.7%)	
72 h–1 week	32 (26.4%)	1 (8.3%)	31 (28.4%)	
> 1 week	14 (11.6%)	5 (41.7%)	9 (8.3%)	
Leucocytes at admission (in 10^9^/l)	12.0, 3.8	12.8, 4.1	11.90, 3.7	*0.492*
CRP at admission (in mg/l)	88.4, 74.7	109.1, 87.6	85.0, 73.0	*0.180*
Number of episode				*0.207*
1	96 (69.1%)	9 (50.0%)	87 (71.9%)	
2	24 (17.3%)	5 (27.8%)	19 (15.7%)	
≥ 3	19 (13.7%)	4 (22.2%)	15 (12.4%)	
Antibiotic therapy				*1.000*
None	1 (0.7%)	0 (0.0%)	1 (0.8%)	
Oral	12 (8.8%)	1 (7.1%)	11 (9.0%)	
Intravenous (± oral)	123 (90.4%)	13 (92.9%)	110 (90.2%)	
Duration of antibiotic treatment (in days)	11.3, 3.4	9.3, 4.6	11.4, 3.3	*0.201*
Hospital stay (in days)	7.3, 5.4	15.6, 9.2	6.0, 2.9	***0.001***

### Statistical analysis and reporting

All calculations were conducted with SPSS® version 26 (IBM, Armonk, NY, USA). Normal distribution was examined by the Shapiro-Wilk test. Categorical variables are presented in frequency tables, and statistical differences between groups are determined by means of chi-square test or Fisher’s exact test. Continuous variables are presented as mean values with standard deviation (SD), with differences between groups assessed by unpaired two-tailed Student’s t test for normal-distributed data and the Mann-Whitney U test for non-normal data. Radiologic variables were deduced from clinical knowledge as possible predictors, and univariable logistic regression was performed. Independent variables reaching a cut-off *p* value of <0.20 were considered for inclusion in a multivariable logistic regression model. For examining unevenly distributed groups, the number of variables included in the final model was restricted to two to avoid overfitting of the model. Thus, all variables identified as significant predictors in the univariable logistic regression models at a cut-off *p* value of 0.20 were subjected to receiver operating characteristics (ROC) curves and area under the curve (AUC) analyses, and the two variables with the highest ROC-AUC values were included in the multivariable regression analysis. Continuous variables were transformed to categorical variables to provide clear cut-off values by determining the optimal cut-off by optimizing the Youden index. For determination of correlation between variables, the Pearson correlation was used if both variables were parametric data and the Spearman correlation if at least one variable was non-parametric. If certain clinical data was missing, cases were only excluded from single analyses, but not from the whole study. Reporting was performed according to the requirements of the STROBE (STrengthening the Reporting of OBservational studies in Epidemiology) checklist.

## Results

### Study population and patient characteristics

Our search routine identified 669 patients with CT scans for suspected diverticulitis, of which 141 patients with acute diverticulitis and contained perforation that were primarily treated conservatively according to current guidelines were included in the analysis (Fig. 1A). The cohort had a mean age of 56.2 (± 14.6) years and consisted of 43% female and 57% male patients. Most included patients were ASA II (48.2%), while 22.7% were ASA I, 24.8% ASA III, 4.3% ASA IV and no patients ASA V or VI. The distribution of comorbidities was inhomogeneous, as 46.1% of patients had no comorbidities, while 34.0% suffered from multiple comorbidities. The majority of 134 patients (95.0%) had no medical immunosuppression (Table 1).

### Disease-related characteristics

At presentation, most patients had signs of systemic inflammation like elevated levels of leucocytes (12.0 ± 3.8 *10^9^/l) and CRP values (88.4 ± 74.7 mg/l). The time between onset of pain and admission was more than 1 week in 12% of patients, between 24 h and 1 week in 51% of patients and less than 24 h in 37% of patients. For 69% of included patients, the admission was their first episode of diverticulitis, while 17% had their second episode, and 14% had already three or more episodes. The majority (90%) of patients were initially treated with intravenous antibiotics, for a mean duration of 11.3 (± 3.4) days. Inpatient treatment was performed for a mean duration of 7.3 (± 5.4) days.

### Image analysis

In the following, the radiologic characteristics of the cohort are described (Table 2). Overall, 18 (12.8 %) patients presented with noticeable liquid abscess formations >1 cm, while 4 patients (2.8%) had abscess formations >4 cm, of which 3 were drained CT-guided, while one was technically not feasible to drain. The length of the inflamed bowel segment was 6.6 (± 3.4) cm. The maximum diameter of the inflamed segment was 2.8 (± 0.5) cm on average, at a maximum wall thickness of 1.9 (± 0.7), leaving a minimal luminal diameter of 8.8 (± 5.3) mm. The area of mesenteric inflammation was 18.6 (± 11.1) cm^2^. Within that area, the tissue was exceeding the radiopacity of non-inflamed intraabdominal fat by 75.5 (± 23.9) HU. This led to a value of 14.2 (± 11.1 HU) * dm^2^ as quantification of total inflammatory affection of the mesentery.

**Table 2:** Overview of the radiologic characteristics of the study population and the subgroups. *p*-values are indicated behind the groups

	Total	Resection within 30 days	No resection within 30 days	*p*
Abscess formation > 1 cm	18 (12.8%)	7 (36.8%)	11 (9.0%)	***0.003***
Abscess formation > 4 cm	4 (2.8%)	1 (5.3%)	3 (2.5%)	*0.354*
Interventional drainage	3 (2.1%)	0 (0.0%)	3 (2.5%)	*1.000*
Length of inflamed bowel segment (in cm)	6.6, 3.4	8.4, 2.9	6.3, 3.4	***0.001***
Max. diameter of inflamed bowel segment (in cm)	2.8, 0.5	2.8, 0.5	2.8, 0.5	*0.937*
Min. lumen of inflamed bowel segment (in mm)	8.8, 5.3	6.6, 4.3	9.1, 5.4	***0.049***
Max. wall thickness of inflamed bowel (in cm)	1.9, 0.7	2.1, 0.7	1.9, 0.7	*0.170*
Area of mesenteric inflammation (in cm^2^)	18.6, 11.1	23.8, 17.4	17.8, 9.6	*0.196*
Density of mesenteric inflammation (in HU)	75.5, 23.9	72.0, 25.3	76.0, 23.0	*0.415*
Total mesenteric inflammation (in HU * dm^2^)	14.2, 11.1	17.2, 15.4	13.7, 10.3	*0.484*

### Correlation between the inflammatory blood markers and quantitative radiologic findings

We detected significant correlations between the blood inflammatory markers (CRP and leucocytes) and the area of mesenteric inflammation and total mesenteric inflammation, respectively. However, leucocyte counts and CRP values did not correlate with the length of the inflamed bowel, while the persisting lumen of the inflamed bowel segment was inversely correlating with leucocyte counts and CRP values. Presence of abscess formations > 1 cm significantly correlated with CRP values (Table 3).

**Table 3:** Correlations between blood inflammatory markers (leucocytes and CRP) and radiologic parameters. As CRP usually increases in a delayed manner, patients with pain onset less than 24 h before presentation were excluded (*N* = 140 patients for leucocytes, *N* = 74 patients for CRP). Pearson correlation was performed for parametric data and Spearman rank correlation for nonparametric data

	Leucocytes at admission (in 10^9^/l)		CRP at admission (in mg/l)	
	Pearson correlation	*p* value	Pearson correlation	*p* value
Length of inflamed bowel segment (in cm)	0.126	*0.135*	0.207	*0.077*
Max. diameter of inflamed bowel segment (in	−0.048	*0.574*	−0.113	*0.339*
Min. lumen of inflamed bowel segment (in mm)	−0.198	***0.018***	−0.313	***0.007***
Max. wall thickness of inflamed bowel (in cm)	0.122	*0.147*	0.174	*0.137*
Area of mesenteric inflammation (in cm^2^)	0.263	***0.002***	0.634	***0.001***
Density of mesenteric inflammation (in HU)	0.048	*0.568*	0.057	*0.632*
Total mesenteric inflammation (in HU * dm^2^)	0.244	***0.003***	0.496	***0.001***
	**Spearman correlation**	*p* value	**Spearman correlation**	*p* value
Abscess formation > 1 cm	0.029	*0.729*	0.365	***0.001***

### Clinical differences in resected patients compared to the non-resected cohort

To determine factors predicting failure of conservative treatment in the acute setting, further analysis was performed in two groups: patients that were resected within 30 days of admission despite initial non-operative management and the remainder. Overall, 19 (13%) patients were resected within 30 days from admission, while 122 patients were not resected within that period (Table 1). Of the resected patients, 12 had sigmoidectomy with primary anastomosis, while 6 patients were treated by Hartmann’s procedure, and 1 patient was treated by laparoscopic lavage. Eight procedures were laparoscopic operations, while 10 were open procedures and one was converted from laparoscopic to open.

Between the groups, we could not find differences in age, sex nor comorbidities or immunosuppression. Interestingly, the early resected patients had significantly higher ASA scores (16% ASA IV, 63% ASA III, 16% ASA II, 5% ASA I) than the group not resected within 30 days (3% ASA IV, 19% ASA III, 53% ASA II, 25% ASA I) (*p* < 0.001). Neither number of episodes nor blood inflammatory markers or type or duration of antibiotic therapy differed between the groups. We found a significant difference regarding the time between onset of pain and admission, 42% of patients had pain for more than 1 week in the group with failed conservative management, while only 8% of patients in the non-resected cohort had pain for longer than 1 week. As expected, the duration of inpatient treatment was longer in the group resected within 30 days (15.6 vs 6.0 days; *p* < 0.001). Of the 19 patients that were resected within 30 days, 16 (84%) were resected within initial hospitalization, while three patients were resected after readmission.

### Radiologic differences

Comparing the radiologic markers of disease, we found significant differences in abscess formation >1 cm and the length of the inflamed bowel segment between the groups (Table 2). Abscesses >1 cm were present in 37% in the resected patients, while only 9% of non-resected patients presented with an abscess >1 cm (*p* < 0.003). Patients in the resected cohort had a greater length of inflamed bowel of 8.4 (± 2.9) cm compared to 6.3 (± 3.4) cm in the non-resected group (*p* < 0.001) and a smaller persisting luminal diameter of 6.6 (± 4.4) cm compared to 9.1 (± 5.4) cm. The maximal bowel diameter, the maximal wall thickness and the area, density and total mesenteric inflammation were not different between the groups.

### ROC analysis and logistic regression

**Fig. 2:**
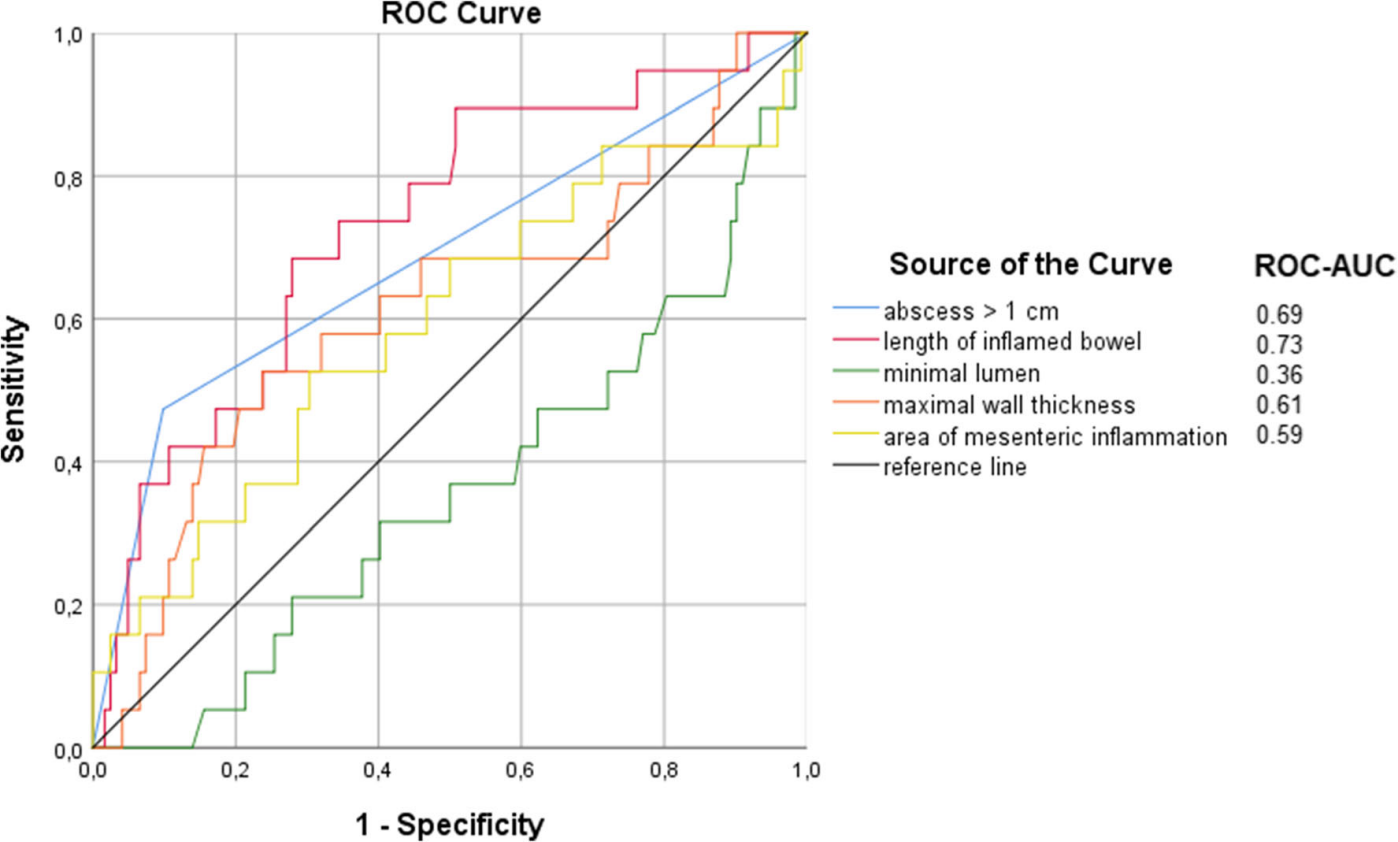
Receiver operating characteristics (ROC) curves and area under the curve (AUC) values for the individual radiological parameters with a significance level *p* < 0.20 in the univariable analysis between the groups (resection within 30 days vs. no resection within 30 days) are plotted

Radiologic disease characteristics as potential predictors for failure of the conservative approach were subjected to logistic regression models. In the first step, univariable logistic regression was performed for all radiologic markers (Table 4): Abscess formations >1 cm (OR 8.3, *p* < 0.001), greater length of inflamed bowel (OR 1.2, *p* < 0.041), lower minimal lumen of the inflamed bowel segment (OR 0.9, *p* < 0.040) and greater area of mesenteric inflammation (OR 1.0, p < 0.040) were significantly associated with resection within 30 days after begin of conservative therapy for diverticulitis with contained perforation. Due to unevenly distributed groups (*n* = 19 patients vs. *n* = 122 patients), only two predictors were included in the final multivariable logistic regression model to avoid overfitting. These were selected by ROC analysis to find the best discriminators, which were the length of the inflamed bowel (ROC-AUC 0.73) and abscess formations >1 cm (ROC-AUC 0.69) (Fig. 2). The length of the inflamed bowel was transposed to a dichotomic variable to provide a clear cut-off value to ease surgical decision-making. The most appropriate cut-off value was determined by optimization of the Youden index and determined at a value of 7 cm (Youden index 0.41). Next, these two variables were subjected to multivariable logistic regression. The multivariable logistic regression model determined length of the inflamed bowel greater 7 cm (*p* < 0.011, OR = 4.1, 95% CI 1.4–12.4) and presence of abscesses >1 cm (*p* < 0.001, OR = 6.6, 95% CI 2.1–20.4) as significant predictors of resection within 30 days. The model was significant (p < 0.001) and had an acceptable coefficient of determination (Nagelkerke’s pseudo-R^2^ = 0.25) (Table 4).

**Table 4:** Univariable logistic regression analysis was performed for all radiologic variables. To avoid overfitting, only two variables were included in the final multivariable logistic regression model. Abscess formation and length of inflamed bowel were selected as they had the highest ROC-AUC values in the ROC analysis. Length of inflamed bowel was transformed into a dichotomic variable prior to inclusion in the multivariable regression model to provide a clear cut-off. A value of 7 cm length of inflamed bowel was determined as optimal discriminator by maximizing the Youden index in the ROC analysis. *OR* odds ratio, *CI* confidence interval

Univariable logistic regression			
Dependent variable: resection within 30 days			
*Variables*	*OR*	*95% CI*	Model *p* value
Abscess formation >1 cm	8.3	*2.8–24.3*	***0.001***
Length of inflamed bowel segment	1.2	*1.1–1.3*	***0.041***
Max. diameter of inflamed bowel segment	0.9	*0.3–2.4*	*0.861*
Min. lumen of inflamed bowel segment	0.9	*0.8–1.0*	***0.040***
Max. wall thickness of inflamed bowel	1.7	*0.8–3.4*	*0.167*
Area of mesenteric inflammation	1.0	*1.0–1.1*	***0.040***
Density of mesenteric inflammation	1.0	*1.0–1.0*	*0.487*
Total mesenteric inflammation	1.0	*1.0–1.1*	*0.242*
Multivariable logistic regression			
Dependent variable: resection within 30 days (Nagelkerke’s R^2^ = 0.25; p < 0.001)			
*Variables*	*OR*	*95% CI*	*p* value
Length of inflamed bowel segment (> 7 cm)	4.1	1.4–12.2	***0.011***
Abscess formation (> 1 cm)	6.6	2.1–20.4	***0.001***

## Discussion

The aim of this study was to determine predictors for failed conservative management for patients suffering from acute diverticulitis with contained perforations (intraabdominal abscess formation of any size and location and/ or pericolic extraluminal gas). These patients represent a population with a high probability of septic progression requiring urgent surgery in the acute setting despite modern multimodal conservative therapy. Nevertheless, even for advanced stages of acute diverticulitis, conservative management was described as being successful in up to 95% [10]. To allow personalized treatment decision on which patients to operate early and which to manage non-operatively during the acute episode is an urgent need. Although historically urgent surgery for acute complicated diverticulitis was performed at a much lower threshold, current trends point towards conservative treatment for as many patients as possible to avoid high mortality rates associated with surgery during the acute episode [10]. We aimed to employ special focus on quantitative radiologic features apart from the established parameters such as distant abscess location, distant air or even generalized purulent or faecal peritonitis. In our cohort, 13% of patients had to be operated within 30 days from initial admission, although a guideline-conform conservative regimen was performed initially. Although study collectives were not completely concordant due to different abscess locations/sizes and air location included, the operation rates of our study are in concordance with current studies on the non-operative management of complicated diverticulitis, which reported rates for urgent surgery in patients with complicated diverticulitis between 5 and 19% [5, 10, 11]. Interestingly, even though immunocompromised patients were described to be prone for failure of conservative treatment, we could not find significant differences in the need for operation in these patients, although the proportion of patients receiving immunosuppression was low in our study cohort [12–14]. We observed significantly more patients with higher ASA scores in the cohort resected within 30 days from admission than in the remainder of patients. Unfortunately, as the ASA score was determined non-standardized by treating physician in the emergency department, we cannot decide whether the scoring was biased by the severe condition of acute diverticulitis and therefore not comparable between the groups. It is not surprising that the group operated within 30 days comprised significantly more patients with more than 1 week between onset of pain and admission as delay of adequate therapy probably allowed progression of intraabdominal sepsis. Surprisingly, about one-third of patients had less than 24 h of pain before admission, while this was the case in only 22% of patients with abscess formations. Still, pain onset might correlate with the time point of perforation, which can occur within 1 day before presentation.

As demonstrated above, we found abscess formations >1 cm and length of the inflamed bowel segment greater than 7 cm as significant predictors for surgery within 30 days despite initial conservative approach. It is not surprising that the presence of abscesses >1 cm was a significant predictor for failure of the antibiotic approach. Abscesses were already described as significant predictors of relapse within 30 days after beginning of conservative therapy of complicated diverticulitis [5, 15]. Still, abscesses only occur in a certain percentage of patients with contained perforations and therefore can only be taken into account for risk evaluation in few cases. Thus, the length of the inflamed bowel segment could be a valuable additional parameter as it can be measured in all diverticulitis patients. In our study, length of inflamed bowel greater than 7 cm was a significant predictor of the failure of conservative treatment. Literature on the correlation between length of the inflamed bowel and disease outcomes, particularly short term, is scarce. One study found an association between length of affected colon and increased risk for disease recurrence [16]. There is another current study by Bates et al. that correlated radiologic findings in diverticulitis patients with the need for operation within a 2-year follow-up interval and demonstrated pericolic fluid collection, free intraperitoneal air, small or large bowel obstruction and colonic fistula to be associated with surgery [6]. Another recent study found that distant intraperitoneal air is the strongest predictor for urgent surgery despite initialized non-operative therapy in a cohort of unselected diverticulitis patients [7]. However, many existing studies include all patients with acute diverticulitis, while we focused on the cohort of patients with contained perforations. These are particularly interesting, as they are primarily treated conservatively according to current guidelines, but in case of failure, surgery can become necessary and be even more challenging. Therefore, identification of risk factors for failure of conservative therapy can indicate in which patients a careful supervision is necessary to avoid further complications through a septic progression of disease. Unfortunately, our cohort did not include enough patients with postoperative complications (e.g. anastomotic leak, surgical site infections) to analyse the influence of failed conservative treatment on postoperative complications.

Being a retrospective cohort study, our study is restricted to its design and immanent limitations, mainly the insufficient separation of the cohorts. Most data is derived from the recordings of the emergency department and therefore is not standardized as the time between onset of pain and admission. Still, we made sure by review of the medical reports that the initial management was conservative in the included patients. Furthermore, the search routine might have missed some patients, for insufficient search terms or external imaging lacking a written report. Furthermore, loss of follow-up cannot be safely excluded, although the short time to the primary endpoint of 30 days may guarantee reliable numbers.

## Conclusions

In summary, we could identify length of the inflamed bowel segment greater than 7 cm and presence of abscess formation >1 cm as prognostic radiologic markers for failure of guideline-conform conservative management of patients with diverticulitis and contained perforations. While most abscesses can be treated interventionally, the length of inflamed bowel was an independent predictor for failure of conservative treatment and should be therefore considered as negative prognostic parameter. Therefore, these patients should be monitored with special caution during conservative treatment, as they have a high risk for septic progression even under multimodal conservative management. Our findings could improve surgical decision-making in those patients, although prospective studies are necessary to confirm the external validity of our findings.
